# Depletion of Arg1-Positive Microglia/Macrophages Exacerbates Cerebral Ischemic Damage by Facilitating the Inflammatory Response

**DOI:** 10.3390/ijms232113055

**Published:** 2022-10-27

**Authors:** Ting Li, Jin Zhao, Hao Gao

**Affiliations:** Gansu Key Laboratory of Biomonitoring and Bioremediation for Environmental Pollution, School of Life Sciences, Lanzhou University, Lanzhou 730000, China

**Keywords:** Arg1, depletion, microglia/macrophages, ischemia, inflammation

## Abstract

Stroke is a serious worldwide disease that causes death and disability, more than 80% of which is ischemic stroke. The expression of arginase 1 (Arg1), a key player in regulating nitrogen homeostasis, is altered in the peripheral circulation after stroke. Growing evidence indicates that ischemic stroke also induces upregulated Arg1 expression in the central nervous system, especially in activated microglia and macrophages. This implies that Arg1 may affect stroke progression by modulating the cerebral immune response. To investigate the effect of Arg1^+^ microglia/macrophages on ischemic stroke, we selectively eliminated cerebral Arg1^+^ microglia/macrophages by mannosylated clodronate liposomes (MCLs) and investigated their effects on behavior, neurological deficits, and inflammatory responses in mice after ischemic stroke. More than half of Arg1^+^ cells, mainly Arg1^+^ microglia/macrophages, were depleted after MCLs administration, resulting in a significant deterioration of motility in mice. After the elimination of Arg1^+^ microglia/macrophages, the infarct volume expanded and neuronal degenerative lesions intensified. Meanwhile, the absence of Arg1^+^ microglia/macrophages significantly increased the production of pro-inflammatory cytokines and suppressed the expression of anti-inflammatory factors, thus profoundly altering the immune microenvironment at the lesion site. Taken together, our data demonstrate that depletion of Arg1^+^ microglia/macrophages exacerbates neuronal damage by facilitating the inflammatory response, leading to more severe ischemic injury. These results suggest that Arg1^+^ microglia/macrophages, as a subpopulation regulating inflammation, is beneficial in controlling the development of ischemia and promoting recovery from injury. Regulation of Arg1 expression on microglia/macrophages at the right time may be a potential target for the treatment of ischemic brain injury.

## 1. Introduction

Ischemic stroke is a common cerebrovascular disease with a high mortality and disability rate. In addition to causing neuronal damage, it is recognized that cerebral ischemia triggers systemic and intracerebral inflammatory responses [1,2,3]. The increase and activation of inflammatory cells and the production and release of inflammatory cytokines are important factors influencing the progression of the disease. Arginase (Arg) has recently been observed to be an emerging key regulator of cellular immunity in the immune system [4]. Although not much research has been done on Arg in stroke, Arg expression has been found to be elevated in both circulatory and central nervous systems. Ischemia-induced Arg release from activated neutrophils in the spleen leads to peripheral immunosuppression [5]. Gene expression and serum activity of Arg are increased in patients with acute ischemic stroke, which is correlated with cerebral infarct volume and stroke severity. Arg is therefore considered a potential biomarker of stroke [6]. In the brain, several studies have found that cerebral ischemia-induced Arg expression is upregulated on infiltrated neutrophils, monocyte-derived macrophages, endothelial cells, resident microglia, and astrocytes [7,8]. Thus it would be important to detect the roles of brain cells expressing Arg during the progression of focal ischemia.

Arg is well-known as a manganese metalloenzyme that hydrolyzes L-arginine to L-ornithine and urea. There are two distinct isoforms of Arg, Arg1, and Arg2, which differ in cellular expression, subcellular localization, and tissue distribution [4,9]. Unlike the mitochondrial enzyme Arg2, Arg1 is expressed in the cytoplasm which plays an important role in the hepatic urea cycle for detoxifying excess nitrogen produced by amino acid metabolism. The deficiency of Arg1 causes severe hyperammonemia, leading to death in mice [10]. The classical role of Arg1 is to regulate NO synthesis in cells by competing with NOS for the substrate L-arginine [11]. In addition, co-localization of Arg1 with ornithine decarboxylase in the cytoplasm directs the Arg metabolite ornithine to polyamines (putrescine, spermidine, spermine) synthesis [12], which plays an important role in cell growth and proliferation and is involved in wound healing, tissue repair, and neurodevelopment. However, the overexpression or increased activity of Arg1 is implicated in a variety of complex diseases. The Arg1/NOS balance is crucial for the pathological development of cardiovascular diseases. After myocardial ischemia-reperfusion, increased expression and activity of Arg1 in coronary endothelial and smooth muscle cells are closely associated with the development of ischemia-reperfusion injury [13,14]. Studies have shown a reduction in myocardial infarct size with the treatment of Arg inhibitors [14,15]. The increase of Arg activity may affect cardiomyocyte survival by reducing NO production and increasing reactive oxygen species formation through decreased eNOS utilization of L-arginine [16]. In diabetes-induced retinal impairment, dysfunction of the vascular endothelium is associated with increased Arg1 protein level and activity, and the vascular dysfunction is markedly blunted by suppressing Arg expression [17]. Additionally, Arg1 also plays a major role in the aging progress by promoting cellular senescence, accelerating vessel stiffness, and vascular endothelial function impairment, which can contribute to atherosclerosis [18].

Besides its fundamental role in the regulation of cellular nitrogen homeostasis, Arg1 has been increasingly shown to be an important inflammation-related factor expressed in the immune system [4]. An important review on Arg in the immune response published by Schneider and Dy in 1985 concluded that activated macrophages can express Arg to consume L-arginine in the microenvironment. This may lead to the suppression of T-cell proliferation and may be implicated in the cytotoxicity of macrophages during the anti-tumor, anti-parasite, and virus response [19]. In the following decades, Arg was found to participate in several inflammatory diseases by reducing nitric oxide synthesis and enhancing fibrosis and tissue regeneration [20,21,22]. In humans, Arg1 is detected abundantly expressed in peripheral blood mononuclear cells, especially in neutrophils, and is released during inflammation [23,24]. Arg1 expression and L-arginine depletion are now crucial components of the immunosuppressive pathway in the mammalian immune system [25]. Moreover, Arg1 has emerged as an important factor in the resolution of the inflammatory response [26,27].

Previous studies have shown that Arg1 expression was upregulated in focal areas after cerebral ischemia, especially in inflammatory cells [7,28]. Infiltrating monocyte-derived macrophages and native brain microglia are important players involved in the neurological immune response after cerebral ischemia and play a vital role in the pathological development of cerebral ischemia [29,30]. However, activated microglia and macrophages exhibit different phenotypes, which may be related to the distinct functions they exercise in complex pathologies. Microglia/macrophages expressing Arg1 are called as M2 type whereas M1 type cells are characterized by iNOS-positive [31,32]. Although the simple dichotomy of microglia/macrophages is controversial, a part of microglia/macrophages are indeed found to upregulate Arg1 expression after cerebral ischemia [33,34,35], and they may act together as a whole to influence the progression of ischemic stroke. In this study, we took MCLs to selectively deplete Arg1^+^ microglia/macrophages via phagocytosis as previous study [33] and assessed the effect of depleting Arg1^+^ microglia/macrophages at the early stage on the cerebral ischemic outcome and neuronal deficits. Our results suggested that Arg1^+^ microglia/macrophages’ deficiency exacerbated stroke-induced injury. Depletion of Arg1^+^ microglia/macrophages exerted an important influence on ischemic injury by altering the immune environment. Ischemia-induced Arg1^+^ microglia/macrophages affect neurological damage by regulating the expression of various inflammatory factors to influence the development of cerebral ischemic injury.

## 2. Results

### 2.1. Dynamics of Arg1^+^ Cells after Acute Ischemic Stroke

**Figure 1:**
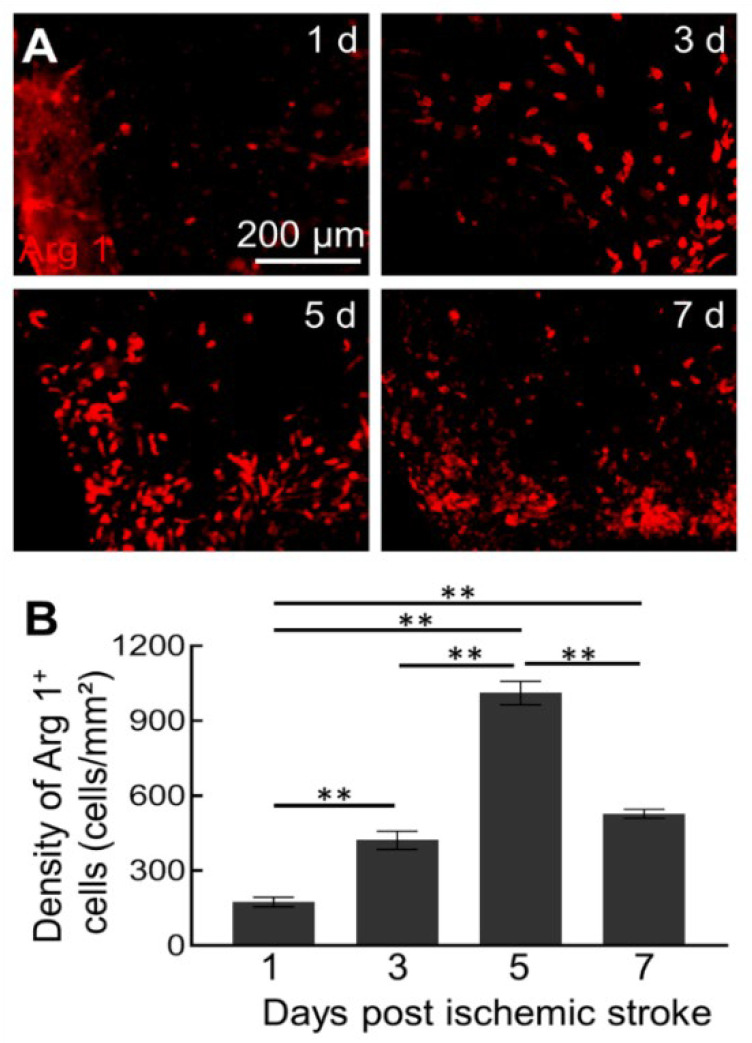
Expression of Arg1^+^ cells within the first week after acute ischemic stroke. (**A**) Distribution of Arg1^+^ cells in the ischemic zone at different days after ischemic stroke. (**B**) The density of Arg1^+^ cells at different days after stroke onset (*n* = 3, F_(3, 8)_ = 116.7, ** *p* < 0.01).

Our previous work has found that acute ischemic stroke-induced strong Arg1 expression in the brain [36]. To investigate the dynamic response of Arg1^+^ cells, we measured cell densities within the first week after ischemic stroke. No Arg1 immunoreactivity was found in the cerebrums of the sham mice without ischemic injury (Supplementary Figure S1). As early as one day after ischemia induction, some Arg1-expressed cells were detected in the lesion site (Figure 1A). The density of Arg1^+^ cells dramatically increased and reached a peak of 1011.5 ± 47.0 cells/mm^2^ on Day 5 after the stroke. After that, the density of Arg1^+^ cells decreased (Figure 1B). 

### 2.2. MCLs Selectively Depletes Arg1^+^ Microglia/Macrophages in the Brain

**Figure 2:**
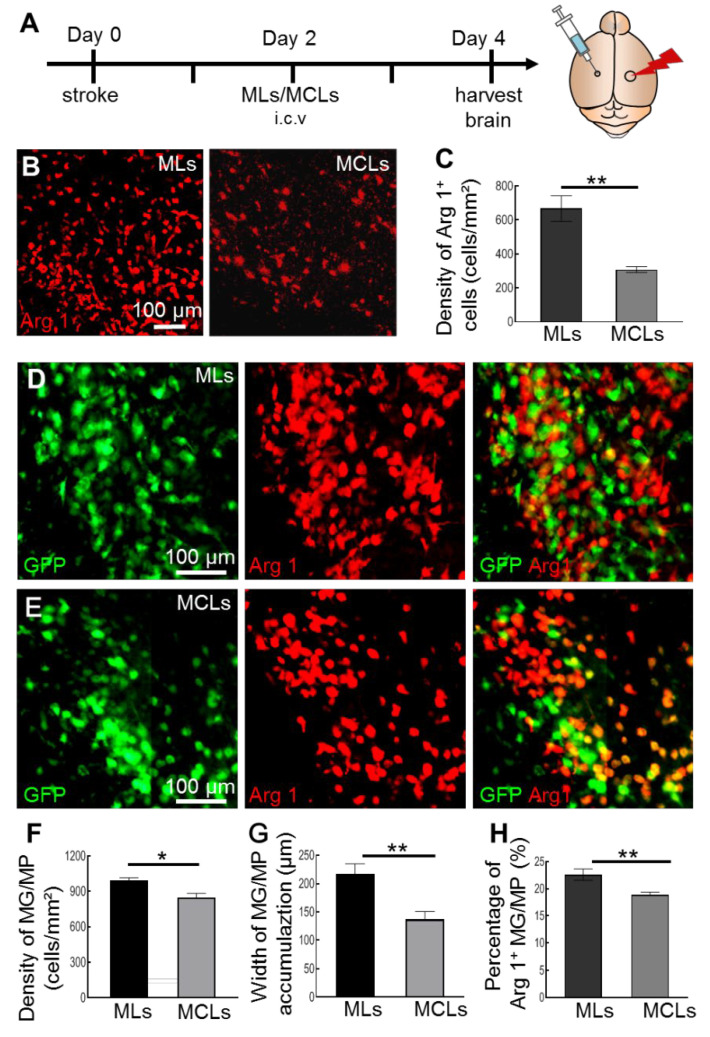
MCLs selectively deplete Arg1^+^ microglia/macrophages four days after ischemic stroke. (**A**) Timeline of drug administration and tissue processing (lightning bolt: ischemic stroke; syringe: intracerebroventricular injection of MLs or MCLs). (**B**) Representative confocal images showing the distribution of Arg1^+^ cells in brain slices administrated with MLs or MCLs after stroke. (**C**) The density of Arg1^+^ cells after MLs or MCLs treatment. MCLs depleted more than half of Arg1^+^ cells after stroke (*n* = 3, ** *p* < 0.01). (**D**) Representative confocal images showing Arg1^+^ microglia/macrophages at the lesion site administrated with MLs after stroke. (**E**) Representative confocal images showing Arg1^+^ microglia/macrophages at the lesion site administrated with MCLs after stroke. (**F**) The density of microglia/macrophages in the accumulation zone after MLs or MCLs treatment (*n* = 3, *p < 0.05). (**G**) The width of microglia/macrophages’ accumulation zone after MLs or MCLs treatment. MCLs caused a significant reduction in activated microglia/macrophages (*n* = 3, * *p* < 0.05). (**H**) The percentage of Arg1^+^ microglia/macrophages after MLs or MCLs treatment. MCLs caused a decrease in Arg1^+^ microglia/macrophages (MG/MP, microglia/macrophages; *n* = 3, ** *p* < 0.01).

Cerebral ischemic stroke causes activation of macrophages and microglia, part of which upregulate Arg1 expression and are important for the development of ischemic pathology [37,38]. To determine the role of Arg1^+^ microglia/macrophages during ischemic injury, selective inhibitor MCLs were administrated into the brain. Since the Arg1^+^ cells increased mainly after Day 1 of the stroke and the MCLs were potent for 2–3 days, we selectively depleted Arg1^+^ microglia/macrophages on Day 2 to Day 4 (Figure 2A). Compared to the vehicle-treated group, the density of Arg1^+^ cells significantly decreased after administration of MCLs, from 667.6 ± 75.4 cells/mm^2^ to 308.5 ± 17.4 cells/mm^2^ (Figure 2B,C). To determine the cell types that were depleted, we checked microglia/macrophages and astrocytes after MCLs treatment. Consistent with a previous study [33], no significant change in the density of GFAP^+^ astrocytes was observed between MLs and MCLs groups after ischemic injury (Supplementary Figure S2). However, microglia/macrophages showed a pronounced reduction in density and width of accumulation zone after MCLs treatment (Figure 2D–G), indicating significant elimination of microglia/macrophage. Meanwhile, 22.6% microglia/macrophages expressed Arg1 in the MLs group while 18.9% in the MCLs group, mainly distributed in the microglia/macrophages accumulation zone (Figure 2D,E,H). These results demonstrated that MCLs specifically and effectively depleted Arg1^+^ microglia/macrophages after stroke.

### 2.3. Depleting Arg1^+^ Microglia/Macrophages Exacerbates Ischemic Injury

**Figure 3:**
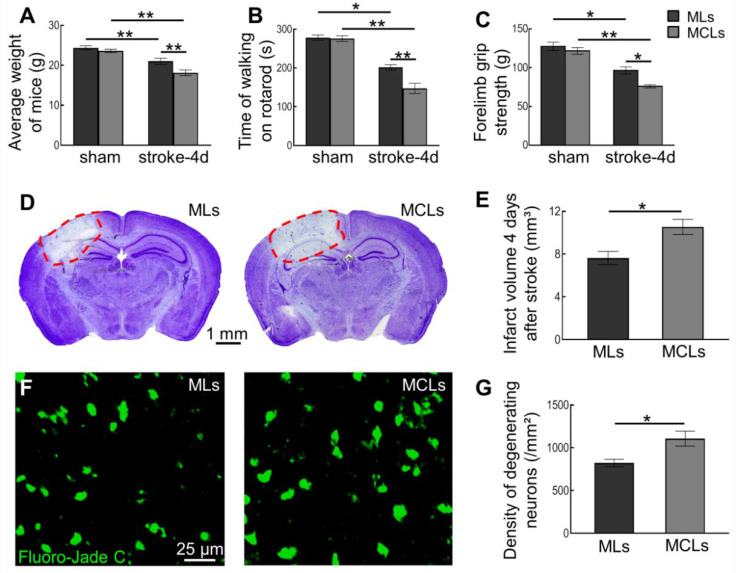
Effects of depleting Arg1^+^ microglia/macrophages on behavioral performance and neuronal deficit in mice 4 days after ischemic stroke. (**A**–**C**) Depletion of Arg1^+^ microglia/macrophages significantly decreased the body weight (**A**), walking time on the rotarod (**B**), and forelimb grip force (**C**) of mice 4 days after stroke. Ischemic stroke itself caused weight loss and worse behavioral performance (*n* = 3, * *p* < 0.05, ** *p* < 0.01). (**D**) Images of Nissl stained brain slices treated with MLs or MCLs after stroke. (**E**) Brain infarct volumes 4 days after stroke. Depletion of Arg1^+^ microglia/macrophages significantly enlarged the infarct size (*n* = 3, * *p* < 0.05). (**F**) Confocal images of Fluoro-Jade C-labeled degenerating neurons in brain slices after MLs or MCLs treatment. (**G**) Densities of degenerating neurons four days after stroke. Depleting Arg1^+^ microglia/macrophages significantly exacerbated neurodegeneration (*n* = 3, * *p* < 0.05).

To evaluate the effect of Arg1^+^ microglia/macrophages depletion on ischemic damage, we first assessed the behavioral performance after ischemic stroke. As expected, ischemic stroke caused badly motor impairment and weight loss (Figure 3A–C). Further reduction in body weight and worse performance on the rotarod test and forelimb grip strength were observed after the depletion of Arg1^+^ microglia/macrophages (Figure 3A–C). Moreover, we also evaluated the infarction and neurodegeneration with Nissl and Fluoro-Jade C stainings, respectively, to reveal neuronal deficit after ischemic stroke. Compared with the vehicle-treated group, our results showed a marked increase in infarct volume (from 7.63 ± 0.62 mm^3^ in the MLs group to 10.54 ± 0.71 mm^3^ in the MCLs group) and density of degenerating neurons (from 821.4 ± 44.5 cells/mm^2^ in the MLs group to 1107.0 ± 86.8 cells/mm^2^ in MCLs group) after MCLs administration (Figure 3D–G and Supplementary Figure S3). These results indicated that depleting Arg1^+^ microglia/macrophages exacerbated the ischemic injury.

### 2.4. Depleting Arg1^+^ Microglia/Macrophages Promotes Inflammation in Ischemic Tissue

**Figure 4:**
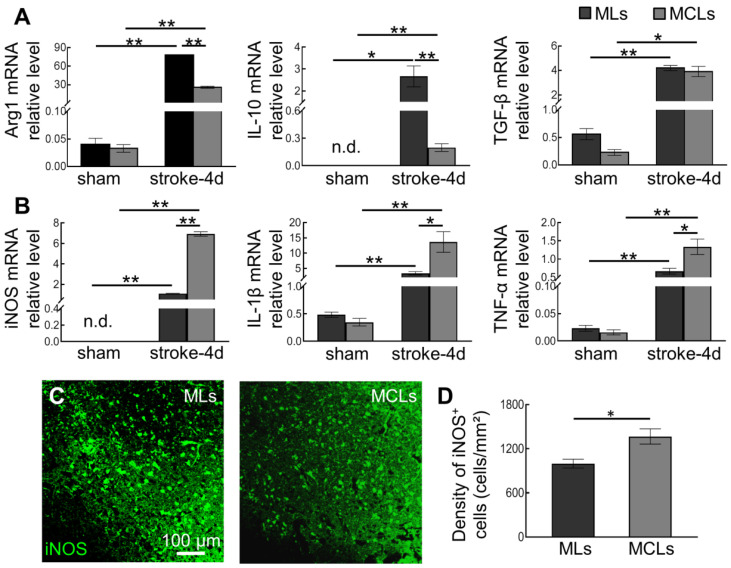
The mRNA expression of key inflammatory factors and iNOS^+^ cells after depletion of Arg1^+^ microglia/macrophages or not. (**A**) Relative mRNA levels of anti-inflammatory factors Arg1, IL-10, and TGF-β. The mRNA expression of Arg1 and IL-10 decreased when Arg1^+^ microglia/macrophages were depleted, while no significant differences were found in TGF-β. However, ischemia itself induced a great increase of inflammatory factors (*n* = 3). (**B**) Relative mRNA levels of pro-inflammatory factors iNOS, IL-1β, and TNF. As Arg1^+^ microglia/macrophages were depleted, the mRNA expression of pro-inflammatory factors significantly elevated four days after stroke. (**C**) Representative confocal images showing iNOS^+^ cells in brain slices administrated with MLs or MCLs after stroke. (**D**) The density of iNOS^+^ cells after MLs or MCLs treatment. MCLs caused a rise of iNOS^+^ cells (*n* = 3, * *p* < 0.05, ** *p* < 0.01).

Consistent with histological results, we detected a dramatic upregulation of Arg1 mRNA levels in focal areas after ischemia induction, and the Arg1 levels significantly dropped after MCLs treatment (from 77.73 ± 0.81 in the stroke-MLs group to 25.80 ± 1.67 in the stroke-MCLs group) (Figure 4A). Given that Arg1 is also considered an anti-inflammatory factor, we detected two more typical factors, IL-10 and TGF-β. Similar to Arg1, ischemia caused remarkable upregulation of IL-10 and TGF-β. After the depletion of Arg1^+^ microglia/macrophages, we found a significant decrease in anti-inflammatory factors IL-10 for 0.92-fold (from 2.66 ± 0.48 in the stroke-MLs group to 0.20 ± 0.04 in the stroke-MCLs group), but no significant change in TGF-β (from 4.20 ± 0.21 in the stroke-MLs group to 3.92 ± 0.41 in the stroke-MCLs group) on Day 4 after stroke (Figure 4A).In contrast, the classic pro-inflammatory factors induced by ischemic stroke exhibited more upregulation after MCLs treatment. Depletion of Arg1^+^ microglia/macrophages lead to a significant increase in mRNA expression of iNOS for 5.29-fold (from 1.10 ± 0.04 in the stroke-MLs group to 6.92 ± 0.21 in the stroke-MCLs group), IL-1β for 3.03-fold (from 3.39 ± 0.55 in the stroke-MLs group to 13.65 ± 3.39 in the stroke-MCLs group), and TNF-α for 1-fold (from 0.67 ± 0.08 in the stroke-MLs group to 1.34 ± 0.21 in the stroke-MCLs group) (Figure 4B). These data suggested that Arg1^+^ microglia/macrophages are the vital producer of anti-inflammatory cytokines after stroke, and suppressed expression of pro-inflammatory cytokines. Furthermore, we measured the density of iNOS^+^ cells in ischemic tissue and found a significant rise after the depletion of Arg1^+^ microglia/macrophages (from 998.4 ± 60.1 cells/mm^2^ in the MLs group to 1367.8 ± 103.5 cells/mm^2^ in MCLs group) (Figure 4C,D). Our previous work proposes iNOS^+^ cells as a key pro-inflammatory player which is detrimental to neuronal survival. Depletion of Arg1^+^ microglia/macrophages significantly shifted the immune microenvironmental profile in the lesion site toward a more severe inflammation, especially increased the iNOS expression in cerebral cells, which is believed to aggravate neural damage.

## 3. Discussion

In general, Arg1 is low expressed in brain tissue. Arg1 expression is upregulated in some cells when individuals undergo tissue injury, such as injury-induced activated inflammatory cells. It is well known that cerebral ischemia leads to intense and persistent inflammation, affecting the progression of pathology [1,39]. Although detailed experimental and clinical information on the role of Arg in ischemic stroke is limited, data suggest an increase in Arg activity after cerebral ischemia [40]. As a key player induced by injury, Arg1 is directly involved in the inflammatory cascade response post-ischemia. We first analyzed the dynamics of all Arg1^+^ cells in brain tissue after acute ischemic stroke. We observed Arg1^+^ cells as early as one day after stroke at the lesion site and peaked on Day 5 after stroke. Arg1^+^ cells were mainly distributed around the core of the lesion. This temporal dynamic of Arg1^+^ cells differs from what we previously reported for iNOS^+^ cells, where the peak of Arg1^+^ cells appeared later than that of iNOS^+^ cells [41]. The relationship between Arg1 and iNOS is particularly close after tissue injury since they compete for the common substrate L-arginine. Our results showed an increase in iNOS^+^ cells after the depletion of Arg1^+^ microglia/macrophages, suggesting that Arg1^+^ microglia/macrophages are important for inhibiting the production of iNOS^+^-derived NO.

As important immune effector cells, a part of activated microglia/macrophages upregulate Arg1 expression after ischemia induction. These cells also upregulate IL-10, CD206, and Ym1 immunoreactivity and are referred to as M2 type “alternative microglia/macrophages’’, while those expressing IL-6, iNOS and CD16/32 are referred to as M1 type “classical microglia/macrophages” [31,32]. However, whether such subtypes actually exist is debatable. In addition to participating in nitrogen homeostasis in the brain through the classical pathway, these microglia/macrophages with highly expressed Arg1 are likely to play an important role in the progression of ischemic injury by influencing the immune environment. It is well proved that interleukins induce Arg1 overexpression through activation of the JAK/STAT6 pathway [42,43,44]. Studies have shown that enhanced Arg activity and polyamine synthesis are involved in the pathogenesis of brain injury, such as Alzheimer’ Disease and Parkinson’s disease, linking to neuronal death [45,46]. However, there is controversy about the role of Arg in cerebral ischemia. Inhibition of systemic Arg expression by inhibitors L-citruline and L-ornithine improves animal behavior and reduces infarction and edema after stroke [47]. A study using Arg2-deficient mice showed larger infarct volume, worse excitotoxic injury, reduced cerebral blood flow, and neurological deficits after cerebral ischemia [48]. However, there is still a lack of research on the role of Arg1 in the brain after cerebral ischemia.

In this work, we selectively depleted cerebral Arg1^+^ microglia/macrophages rather than inhibiting systemic Arg expression. The lack of specificity in current Arg1 inhibitors and the limited life span of Arg1 knockout mice present considerable disadvantages [10,49]. Indeed, the function of Arg1 after ischemia may be organ-dependent and cell-specific, thus it is necessary to study them differently. As a key player in brain inflammation, microglia/macrophages respond strongly to brain injury and directly produce various inflammatory factors. Focusing on the role of Arg1^+^ microglia/macrophages in cerebral ischemia, we depleted Arg1^+^ microglia/macrophages with selective inhibitors MCLs. According to our previous study, a large number of activated microglia and a few infiltrated macrophages accumulated in the lesion site during the first 4 days of cerebral ischemia [29]. In our paradigm, a part of Arg1^+^ microglia/macrophages was depleted after MCLs treatment. We consider several possibilities for these residual Arg1^+^ microglia/macrophages. On the one hand, to reduce the mechanical manipulative damage to the mice caused by ventricular injection, we injected MCLs once on the second day after stroke. Since the effect of MCLs lasts for more than 48 h, it was the Arg1^+^ microglia/macrophages on the third and fourth day after stroke that were cleared. Thus Arg1^+^ microglia/macrophages from the first two days of stroke were retained. On the other hand, we suspect that some Arg1^+^ microglia/macrophages survived because of their inherent low sensitivity to MCLs. Therefore, the effect of Arg1^+^ microglia/macrophages on cerebral ischemia may be even underestimated.

In contrast to the result of a previous study in which Arg inhibitors had a protective effect on ischemic brain injury, we found an aggravated ischemic injury after the elimination of Arg1^+^ microglia/macrophages [47]. Our results showed that the depletion of Arg1^+^ microglia/macrophages led to larger infarct volume and worse neurodegeneration. Meanwhile, significant upregulation of pro-inflammatory factors and downregulation of anti-inflammatory factors were detected in ischemic tissue, indicating that Arg1^+^ microglia/macrophages have a pronounced anti-inflammatory effect. Many studies have shown that pro-inflammatory cytokines directly or indirectly affect neuronal survival and ischemic injury. iNOS-derived NO induces mitochondrial damage to trigger neuronal apoptosis, and inhibiting iNOS expression and activity is beneficial in reducing the damage caused by stroke [50,51]. IL-1β exerts an essential role in ischemic stroke by aggravating the BBB dysfunction and inflammatory response, as well as activating the apoptosis of damaged cells [52]. In an ischemic stroke setting, TNF-α is involved in inflammatory and prothrombotic events. Inhibition of TNF-α decreases activation of immune cells, reduces cerebral infarction, and improves functional outcomes [53,54]. Upon depletion of Arg1^+^ microglia/macrophages, the exacerbation of neuronal damage may be attributed to increased inflammatory response. It demonstrates that Arg1^+^ microglia/macrophages play an important role in regulating the immune microenvironment of the brain, acting as a neuroprotective agent in ischemic stroke. The Arg1^+^ microglia/macrophages alleviate ischemic injury by releasing anti-inflammatory factors and inhibiting the production of pro-inflammatory factors, thereby influencing the pathological development of ischemic stroke. 

The role of microglia in the development of brain injury following stroke has been controversial. Some studies have suggested that microglia protect against neural injury [55,56], while others have shown that microglia promote the exacerbation of stroke injury [36,57]. One of our previous studies has shown that iNOS^+^ microglia/macrophages exacerbate neuronal degeneration by facilitating inflammatory response [41]. In the present study, our results showed that Arg1^+^ microglia/macrophages alleviated ischemic injury through anti-inflammatory effects. A recent study using single-cell analysis of central nervous system tissues revealed multiple subtypes of microglia with distinct molecular characteristics [58]. These suggest that the role of microglia is the result of a combined effect of diverse subsets. Stroke leads to dynamic changes in microglia with different phenotypes. The balance among various microglia affects the inflammatory microenvironment in the lesion site, which contributes to the pathological development. Therefore, it is necessary to focus on microglia with various phenotypes during the investigation of microglia behavior and function.

## 4. Materials and Methods

### 4.1. Animals

CX3CR1^GFP/+^ mice expressing green fluorescent protein (GFP) in monocytes and microglia (JAX #005582) were purchased from the Jackson Laboratory. All transgenic mice and C57BL/6 mice aged 3–4 months were bred in-house under standard environmental (temperature 22 ± 2 °C, humidity 55 ± 10%, light-dark circle 12:12, with nutritional food and water available ad libitum. All animal experiments were performed following ethical approval by the Ethics Committee of Lanzhou University, China.

### 4.2. Photothrombotic Ischemic Stroke Model

A photothrombotic ischemic stroke model was induced according to the previously described method [41]. Briefly, mice were anesthetized by intraperitoneal injection of a ketamine-xylazine mixture (20 mg/mL ketamine and 2 mg/mL xylazine, 0.1 mL/20 g mouse). A small cranial window ~40 μm in thickness was thinned over the right somatosensory cortex (−2 mm from bregma and −2 mm later) with a high-speed drill. Photosensitive rose bengal (0.03 mg/g mouse, Sigma-Aldrich, MO, USA, Cat# R3877) was intravenously injected from the tail vein, and then the thinned skull was exposed under 535 ± 25 nm green light to activate the dye to induce focal ischemia. Mice in the sham group were subjected to an identical procedure except for fluorescence excitation to rose bengal.

### 4.3. Selective Depletion of Arg1^+^ Microglia/Macrophages after Ischemia

Mannosylated clodronate liposomes (MCLs) recognize and bind to M2-type microglia/macrophages which upregulated mannose receptors [33]. MCLs trigger apoptosis in M2-type microglia/macrophages via clodronate-mediated iron depletion and disruption of intracellular ATP metabolic homeostasis within 2–3 days. Since M2-type microglia/macrophages simultaneously upregulate Arg1, we used MCLS to deplete Arg1^+^ microglia/macrophages after ischemia. Drug administration procedures were modified from Miron, V. E. et al. [33]. On the second day after stroke surgery, 4 µL mannosylated liposomes (MLs, as vehicle, Encaspsula NanoSciences) or MCLs (Encaspsula NanoSciences, Brentwood, USA) were intracerebroventricularly injected and depletion of Arg1^+^ microglia/macrophages was assessed 4 days post-stroke.

### 4.4. Infarct Volumetry 

Mice were anesthetized by an overdose of urethane and perfused transcardially with PBS followed by 4% paraformaldehyde. The brains were harvested and fixed in paraformaldehyde for 24 h and then sectioned into 30 μm coronal slices by a vibrating microtome. For Nissl staining, every fourth brain slice was collected and stained with cresyl violet. The infarcted area of each slice was assessed by Image J software version 1.53t (NIH, USA). The Loihl method was employed to correct for cerebral edema [59]: [infarcted area] = [area of the contralateral hemisphere] − [non-infarcted area of the ipsilateral hemisphere]. The total infarct volume was determined by multiplying the infarcted area of each slice by the distance between sections (120 μm).

### 4.5. Histochemical Stainings

For immunohistochemistry and Fluoro-Jade C staining, three brain slices with the injured area of each mouse were randomly picked for each staining. The primary antibodies were used as follows: rabbit-anti-Arg1 (1:300, Boster, Wuhan, China, Cat# BA0056), mouse-anti-iNOS (1:300, BD Biosciences, Franklin Lakes, USA, Cat# 610328), and rabbit-anti-GFAP (marker for astrocytes, 1:300, Boster, Cat# BA3796-2). Followed by appropriate secondary antibodies, the sices were imaged under an epifluorescence (Olympus, BX51) or a confocal microscope (Olympus, Japan, FV1000). Three rectangles (200 μm × 100 μm) were randomly selected on the borders of the immunoreactive region of each slice, inwards from the interface between staining-positive and –negative tissue. Then the cell densities were analyzed by ImageJ software. To assess neurodegeneration, Fluoro-Jade C staining was performed as described previously. In brief, randomly selected slices were placed in an ethanol gradient and then incubated in a 0.06% potassium permanganate solution. Slices were immersed in 0.0001% Fluoro-Jade C solution (Merck, Billerica, USA, Cat# AG325) to stain the degenerative neurons. The slices were dried and cleared in xylene and then mounted in DPX. Three rectangles (200 μm × 100 μm) were randomly selected on the borders of the infarct region, where the density of FJC-positive cells was measured with Image J.

### 4.6. Behavioral Tests

The forelimb grip strength test and rotarod test were performed to evaluate the motor ability of mice as described previously [41]. All behavioral performance and body weight were recorded on the day when each mouse’s brain was harvested. To assess muscle strength, the forelimbs of the mouse were placed on a triangle frame attached to a sensitive force transducer. The tail of the mouse was dragged backward until the forelimbs were released from the metal bar of the triangle frame. The maximal power of forelimb grip strength was recorded using the Biological Data Acquisition and Analysis System (Taimeng BL-420F, Chengdu, China). The grip strength of each mouse was measured five times and the mean value was taken for statistics. To assess the motor coordination and balance of the mouse, the rotarod test was carried out on a rotarod treadmill apparatus (Taimeng ZB-200, Chengdu, China). Pre-training was performed on the apparatus for three days to select eligible mice and 1-2 mice in each experimental group that could not keep walking on the rotarod (accelerated from 10 rpm to 30 rpm within 5 min) for at least 300 s were discarded. The time spent walking on rotarod was recorded three times for each mouse and the mean was used for statistical analysis.

### 4.7. Quantitative Real-Time Polymerase Chain Reaction (qRT-PCR)

Total RNA was isolated from the injured area of the brain using an RNAprep pure Tissue Kit (TIANGEN, DP431), and reverse transcription was performed using the PrimeScript™ RT reagent Kit with gDNA Eraser (TAKARA, Cat# RR047A). Subsequently, qRT-PCR procedures were carried out using a commercial mix (SYBR@ Premix Ex TaqTMII, TAKARA, Cat# RR820A) on the Real-Time PCR system (Bio-Rad, Hercules, CA, USA) according to the manufacturer’s protocols. Data were normalized to GAPDH and the fold change between different transcript levels was calculated by the ΔΔCT method. The primers of inflammatory factors were shown in Table 1.

### 4.8. Statistical Analysis

Image and data analysis was performed in a blinded manner. Statistical analysis was conducted with the GraphPad Prism 7.0 software and results were expressed as mean ± standard errors of the mean (sem). The dynamics of Arg1^+^ cells were compared using one-way ANOVA, the behavioral results and qRT-PCR data were compared using two-way ANOVA. Tukey’s test was performed for multiple comparisons. Single comparisons of infarct volumetry and histological data were made using Student’s *t*-test. (* *p* < 0.05, ** *p* < 0.01). 

## 5. Conclusions

In this work, we took MCLs to deplete ischemia-induced Arg1^+^ microglia/macrophages in the brain. After the depletion of Arg1^+^ microglia/macrophages, we found worse neurodegeneration and stroke outcome in mice. In the meantime, significant downregulation of anti-inflammatory factors and upregulation of pro-inflammatory factors were detected which indicated an increased inflammatory response at the lesion site. Our findings suggest that Arg1^+^ microglia/macrophages may alleviate neuronal injury by suppressing inflammatory responses. Therefore, the Arg1^+^ microglia/macrophages can be considered as a target subpopulation that could be regulated in ischemic stroke. Promoting the expression of anti-inflammatory factors in this subpopulation is beneficial to ischemic injury, implying a potential approach to treat neurological injury.

## Figures and Tables

**Table 1:** Primer sequences of inflammatory factors.

Gene	Forward	Reverse
*GAPDH*	5′-TGAACGGGAAGCTCACTGG-3′	5′-TCCACCACCCTGTTGCTGTA-3′
*iNOS*	5′-CAAGCACCTTGGAAGAGGAG-3′	5′-AAGGCCAAACACAGCATACC-3′
*IL-1β*	5′-CCTCGTGCTGTCGGACCCATA-3′	5′-CAGGCTTGTGCTCTGCTTGTGA-3′
*TNF-α*	5′-GACGTGGAACTGGCAGAAGA-3′	5′-ACTGATGAGAGGGAGGCCAT-3′
*Arg1*	5′-TCACCTGAGCTTTGATGTCG-3′	5′-CTGAAAGGAGCCCTGTCTTG-3′
*IL-10*	5′-CCAAGCCTTATCGGAAATGA-3′	5′-TTTTCACAGGGGAGAAATCG-3′
*TGFβ*	5′-GGCGATACCTCAGCAACCG-3′	5′-CTAAGGCGAAAGCCCTCAAT-3′

## Supplementary Materials

Supporting information can be downloaded at: https://www.mdpi.com/article/10.3390/ijms232113055/s1.
