# Supplementary material for: Depletion of Arg1-Positive Microglia/Macrophages Exacerbates Cerebral Ischemic Damage by Facilitating the Inflammatory Response
Source: Int J Mol Sci. 2022 Oct 27;23(21):13055. doi: 10.3390/ijms232113055 (PMC9655877; doi:10.3390/ijms232113055)
Supplement: Supplementary file 1 [file ijms-23-13055-s001.zip › ijms-1952524-supplementary.pdf]

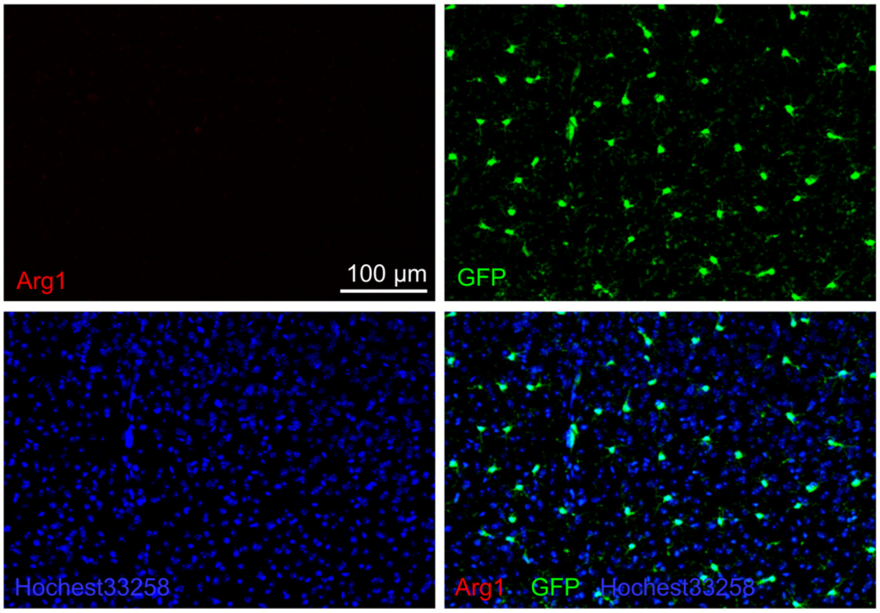

Figure S1. Arg1 expression in the healthy brain

No Arg1 immunoreactivity (red) was observed in the contralateral brain without ischemic insult. Microglia (green) distribute evenly in the cerebral cortex of healthy CX3CR1$^{GFP/+}$ mice. Cell nuclei were marked with Hochest 33258.

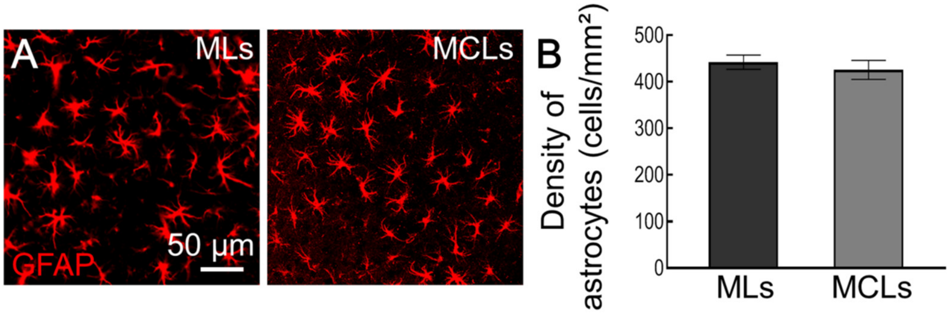

Figure S2. Distribution of astrocytes after stroke

(A) Representative images of brain slices showing GFAP^{+}-astrocytes with MLs or MCLs after stroke. (B) The density of astrocytes after MLs or MCLs treatment. MCLs had no effect on astrocyte density (n = 3).

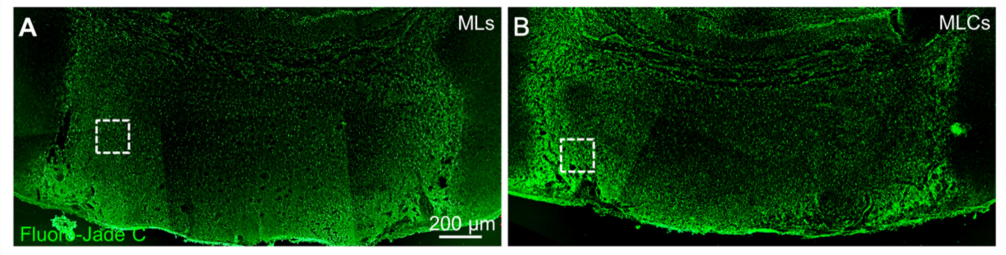

Figure S3. Distribution of degenerative neurons at the lesion site after stroke

(a) Brain slice stained with Fluoro-Jade C treated with MLs at 4 days after stroke. (B) Brain slice stained with Fluoro-Jade C treated with MCLs at 4 days after stroke. The higher magnification images of the white box regions are respectively shown in Fig. 3 F.
